# Long non-coding RNA CRNDE regulates the growth and migration of prostate cancer cells by targeting microRNA-146a-5p

**DOI:** 10.1080/21655979.2021.1935402

**Published:** 2021-07-07

**Authors:** Dewang Fu, Li’e Zang, Zhaowei Li, Chenghui Fan, Huamao Jiang, Tongyi Men

**Affiliations:** a Department of Urology Surgery, Shandong Qianfoshan Hospital, Cheeloo College of Medicine, Shandong University, Jinan, China; b Department of Urology Surgery, The First Affiliate Hospital of Jinzhou Medical University, Jinzhou, China

**Keywords:** Prostate cancer, lncRNA crnde, miR-146a-5p

## Abstract

The function of lncRNA CRNDE and its role in prostate cancer (PC) remains unclear. The aim of this study was to determine the expression level of lncRNA CRNDE in PC tissues and to elucidate its role in PC. The expression levels of lncRNA CRNDE were measured by quantitative reverse transcription polymerase chain reaction. The role of lncRNA CRNDE in PC cells was studied using loss-of-function assays *in vitro*. Cell proliferation, migration, invasion, and apoptosis were assessed via Cell Counting Kit-8, colony formation, flow cytometry, wound healing, and transwell chamber assays. A luciferase reporter assay was used to characterize the interaction between lncRNA CRNDE and miR-146a-5p. In PC tissues, the expression level of lncRNA CRNDE was upregulated. Moreover, knockdown of lncRNA CRNDE suppressed PC cell proliferation and migration and induced apoptosis *in vitro*. miR-146a-5p was verified as a direct target of lncRNA CRNDE. Moreover, the inhibition of miR-146a-5p partially counteracted the effects of lncRNA CRNDE on PC cell proliferation, migration, and invasion. In conclusion, lncRNA CRNDE may serve as a cancer promoter in PC by targeting miR-146a-5p. Therefore, lncRNA CRNDE could be a promising target for the clinical treatment of PC.

## Graphical abstract

**Figure:**
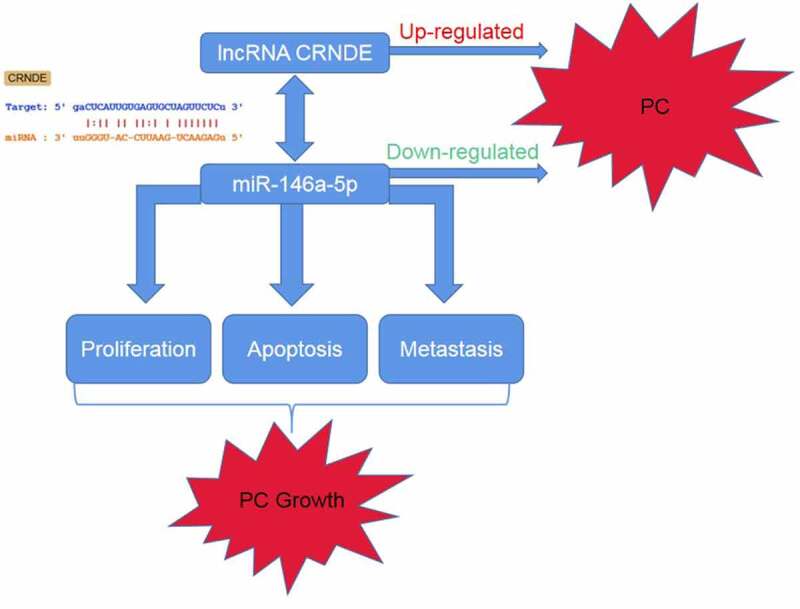

## Introduction

Prostate cancer (PC) is a highly lethal malignant cancer of the urinary system. It is the second leading cause of cancer-associated deaths worldwide in men [1–3]. At present, great advances have been achieved in surgery, chemotherapy, and adjuvant therapy. However, PC remains a noteworthy public health burden due to its high mortality and the prognosis of PC patients remains unsatisfactory. Moreover, the specific molecular mechanisms underlying the incidence and development of PC remain unknown.

Long non-coding RNAs (lncRNAs) are functional transcripts of more than 200 nucleotides in length [4]. Recently, growing evidence has shown that lncRNAs are involved in the development of cancers as oncogenes or cancer suppressor genes, providing new therapeutic targets for cancer treatment [5–7]. Many lncRNAs are abnormally expressed in various tumors, including PC. For instance, recent research has shown that lncRNA MALAT1 promotes the proliferation and metastasis of aggressive pancreatic cancer through autophagy stimulation [8]. According to Wang et al., lncRNA PVT1 promoted the growth of cervical squamous cell carcinoma [9]. Furthermore, lncRNA FER1L4 has been found to suppress the growth and invasion of cancer cells in esophageal squamous cell carcinoma [10]. In addition, lncRNA MALAT1 acts as a sponge for miR-200 c in endometrial carcinoma [11]. However, there is still insufficient information about the precise function of lncRNA CRNDE and the underlying mechanism of its role in PC regulation.

The aim of this study was to explore the specific mechanism of lncRNA CRNDE on the growth and metastasis of PC. We hypothesized that lncRNA CRNDE served as a cancer promoter in PC through targeting miR-146a-5p, and lncRNA CRNDE might represent a promising goal for the clinical PC treatment.

## Materials and methods

### Human samples

Tumor tissues and paired adjacent normal tissues were obtained from the Qianfoshan Hospital affiliated with Shandong University. Tissue samples were obtained from 25 PC patients who underwent surgery at the hospital between May 2017 and September 2018. The samples were frozen in liquid nitrogen and stored at −80°C until further use.

### Ethics statement

This research was approved by the ethical committee of Qianfoshan Hospital affiliated with Shandong University (approval number 20,170,423). Informed consent was obtained from all patients.

### Cell culture and transfection

Human PC cell lines LNCaP, PC3, DUL145, and VCaP, as well as normal prostate epithelial cells (RWPE-1), were purchased from Cell Bank, Shanghai Institutes for Biological Sciences (Shanghai, China). The cells were cultured in RPMI-1640 medium (Gibco, Carlsbad, CA, USA) supplemented with 10% fetal bovine serum (Gibco) at 37°C with 5% CO_2_. SiRNA targeting lncRNA CRNDE (siRNA 1#, siRNA 2#) and si-NC, miR-146a-5p inhibitor and inhibitor NC were synthesized by GenePharma (Shanghai, China). PC3 cells were transfected with siRNA lncRNA CRNDE, siRNA lncRNA CRNDE + miR-146a-5p inhibitor, or a negative control (siRNA NC) using Lipofectamine 2000 (Invitrogen, Carlsbad, CA, USA). Briefly, the PC3 cells were cultured in F12 + 10%FBS medium for 24 h until the cell confluence reached 80%. Then the cells in logarithmic growth phase were collected to be digested by pancreatin, inoculated into 6-well culture plates and added with appropriate amount of virus for infection. After transfection, the cells were further cultured 72 h for further analysis. The siRNA and inhibitor sequences are as follows:

siRNA 1#: 5ʹ-GUGCUCGAGUGGUUUAAAUTT-3ʹ (sense),

5ʹ-AUUUAAACCACUCGAGCACTT-3ʹ (anti-sense)

siRNA 2#: 5ʹ-GGGUAUUCCUGUUUAUAGATT-3ʹ (sense)

5ʹ-UCUAUAAACAGGAAUACCCTT-3ʹ (anti-sense)

si-NC: 5ʹ-GCGACGAUCUGCCUAAGAUTT-3ʹ (sense)

5ʹ-AUCUUAGGCAGAUCGUCGCTT-3ʹ(anti-sense)

inhibitor: Sense 5ʹ-AACCCAUGGAAUUCAGUUCUCA-3ʹ

inhibitor NC: Sense 5ʹ-CAGUACUUUUGUGUAGUACAA-3ʹ

### Bioinformatic analysis

The target miRNA and the binding sites of lncRNA CRNDE was predicted through the online software starBase (http://hopper.si.edu/wiki/mmti/Starbase), DIANA (http://carolina.imis.athena-innovation.gr/diana_tools/web/index.php?r=site%2Ftools) and miRDB (http://mirdb.org/index.html).

### Real-time PCR (RT-PCR)

Total RNA was isolated from cells and tissues using TRIzol Reagent (Invitrogen) according to the manufacturer’s instructions. RT-PCR was conducted using the ABI 7300-fast Real Time PCR system (Applied Biosystems) with SYBR Green PCR Kit (Qiagen). Gene expression levels were calculated using the 2^−ΔΔCT^ method according to Khan et al [12]. GAPDH and U6 were used as standardized internal controls.

### Western blot assay

The western blot was conducted as described by Pillai et al [13]. RIPA lysis buffer was used to extract proteins from the cells. Protein concentrations were determined using the BCA Protein Assay Kit (Beyotime, Beijing, China). Then, the protein was separated by 10% sodium dodecyl sulfate polyacrylamide gel electrophoresis (SDS-PAGE) and transferred onto polyvinylidene fluoride (PVDF) membranes. The membranes were blocked with 5% nonfat milk in TBST at room temperature for 1 h and then incubated with primary antibodies overnight at 4°C. Subsequently, the membranes were washed three times with TBST and probed with HRP-conjugated secondary antibody. The protein blots were detected using ECL detection reagent (Thermo Scientific, CA, USA) and analyzed using ImageJ software (version 1.8.0; National Institutes of Health, Bethesda, MD, USA).

### Cell counting kit-8 (CCK-8) assay

The viability of PC3 cells was determined via CCK-8 assay according to previous study [14]. Briefly, cells were seeded in 96-well plates at a density of 1 × 10^4^ cells per well. After 12 h, the cells were transfected and incubated for an additional 0, 24, 48, or 72 h. Subsequently, 10 μL of CCK-8 solution was added to each well. After another 3 h, a microplate reader was used to record the optical density (OD) at 450 nm.

### Colony formation assay

The colony formation assay was performed as described by Zhang et al [15]. After transfection for 48 h, PC3 cells were seeded in 24-well plates and cultured. Next, the cells were fixed with 4% ethanol and stained with 1% crystal violet. Finally, a microscope was used to image the colonies.

### Flow cytometry assay

The Annexin V-FITC kit (Beyotime Biotechnology, Shanghai, China) [16] was used to assess the role of lncRNA CRNDE in PC3 cell apoptosis. In brief, cells were seeded in 24-well plates at a density of 1 × 10^4^ cells per well, cultured for 48 h, washed with PBS, and resuspended in 500 μL binding buffer. The cells were then incubated for 15 min with Annexin V-FITC (5 μL) and PI (10 μL). Finally, a flow cytometer (BD Biosciences, USA) was used to determine the number of apoptotic cells.

### Transwell assay

A transwell chamber (BD Biosciences) was used to assess cell migration and invasion according to a previous study [17]. The cells in serum-free medium were placed in the upper compartment on a Matrigel-coated membrane for the migration assay or on an uncoated membrane for the invasion assay, while the lower compartment was filled with culture medium containing serum. After 24 h of incubation in the serum-free medium, cells on the upper part of the membrane were wiped off, and the membranes were stained with crystal violet. Then, migration and invasion were quantified by using a microscope to count the stained cells in five different fields (200× magnification).

### Luciferase reporter assay

The target gene of lncRNA CRNDE was predicted to be miR-146a-5p by TargetScan, miRBase, and Picta. lncRNA CRNDE 3ʹ-UTR wild type (WT) or mutant (MUT) plasmids together with miR-146a-5p mimic or NC mimic were co-transfected into the PC3 cells. After 24 h, luciferase activity was detected using the Dual-Luciferase Reporter System kit (Promega Corporation) as described by Unal et al [18].

## Statistical analysis

Data analysis was performed using GraphPad Prism 5.0. Values are expressed as the mean ± standard deviation. Student’s *t*-test was used to analyze the differences between the two groups. One-way ANOVA was used for comparisons among three or more groups. Statistical significance was set at P < 0.05.

## Results

In this research, the expression levels of lncRNA CRNDE in the PC tissues was increased. Knockdown of lncRNA CRNDE suppresses cell proliferation migration, invasion as well as promotes cell apoptosis in PC. At the molecular level, our results validated that miR-146a-5p was a direct target of lncRNA CRNDE. Ultimately, our findings confirmed that lncRNA CRNDE served as a cancer promoter in PC through targeting miR-146a-5p.

### High expression of lncRNA CRNDE was observed in PC tissues and cells

**Figure 1.**
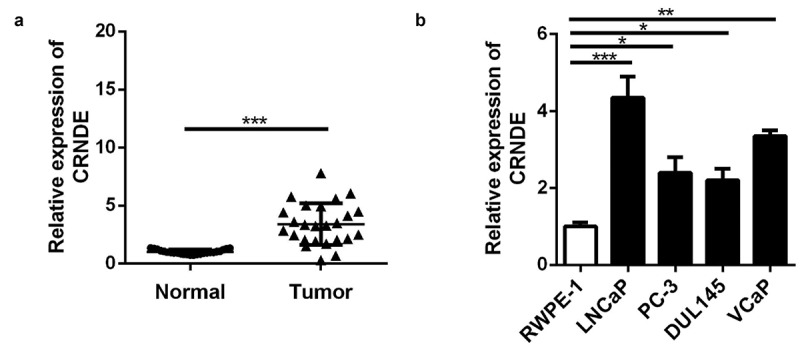
The lncRNA CRNDE expression was up-regulated in PC. RT-PCR analysis was performed to measure the expression of lncRNA CRNDE in PC tissues (a) and cells (b).*p < 0.05, **p < 0.01, ***p < 0.001. Each cell experiment was repeated for 3 times

First, in order to explore the role of lncRNA CRNDE in PC, qRT-PCR was used to assess lncRNA CRNDE mRNA expression in PC (n = 25) and corresponding (n = 25) tissues. The results showed that in comparison with that in corresponding tissues, lncRNA CRNDE expression in PC tissues was upregulated (Figure 1a). In addition, the expression of lncRNA CRNDE was upregulated in all four PC cell lines compared to that in RWPE-1 cells (Figure 1b).

### miR-146a-5p was identified as a target of lncRNA CRNDE

**Figure 2.**
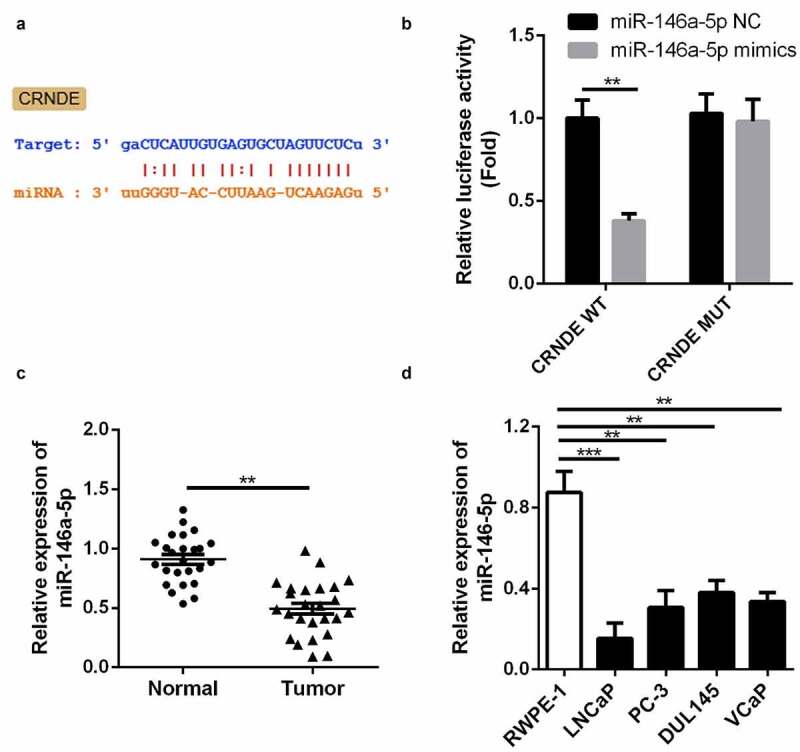
miR-146a-5p is a target of lncRNA CRNDE. (a) The predicted lncRNA CRNDE binding site in the miR-146a-5p 3ʹ-UTR . (b) Relative luciferase activity of cells after co-transfection with wild type (WT) or mutant (Mut) lncRNA CRNDE 3′-UTR reporter genes and miR-146a-5p mimics. (d) miR-146a-5p was up-regulated in PC tissues. (D) miR-146a-5p was up-regulated in all 4 PC cells in comparison with the RWPE-1 cells. **p < 0.01, ***p < 0.001. Each cell experiment was repeated for 3 times

We employed three publicly available target prediction tools to predict the targets of lncRNA CRNDE. miR-146a-5p was identified as a target via StarBase and subjected to further analysis (Figure 2a). As shown in Figure 2b, luciferase activity decreased in cells co-transfected with WT lncRNA CRNDE 3ʹ-UTR and miR-146a-5p mimic compared with that in the control group. Moreover, the cells co-transfected with MUT lncRNA CRNDE 3ʹ-UTR exhibited no change in luciferase activity. qRT-PCR was used to measure the expression of miR-146a-5p in PC and corresponding tissues (Figure 2c); decreased expression of miR-146a-5p was observed in PC tissues. These results demonstrate that miR-146a-5p may be a direct target of lncRNA CRNDE. Moreover, the expression of miR-146a-5p was downregulated in all four PC cell lines compared to that in RWPE-1 cells (Figure 2d).

### Knockdown of lncRNA CRNDE inhibited proliferation of PC cells

**Figure 3.**
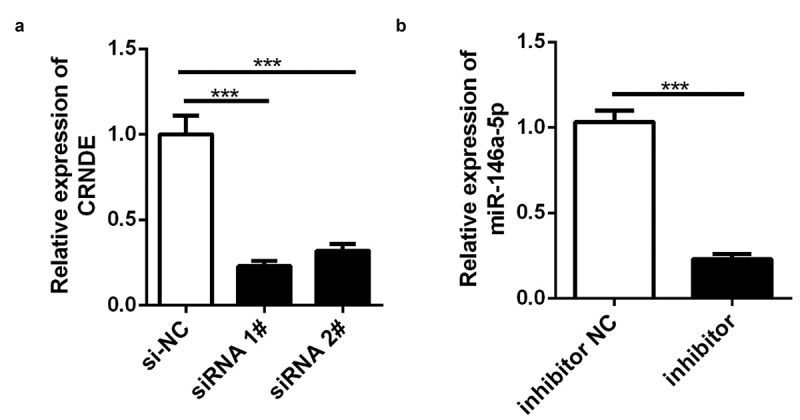
SiRNA and inhibitor down-regulated the expressions of CRNDE and miR-146a-5p. (a) The expression of CRNDE was detected by qRT-PCR after siRNA 1# and siRNA 2# transfection. (b) The expression of miR-146a-5p was measured by qRT-PCR after inhibitor transfection. ***p < 0.001. Each cell experiment was repeated for 3 times

**Figure 4.**
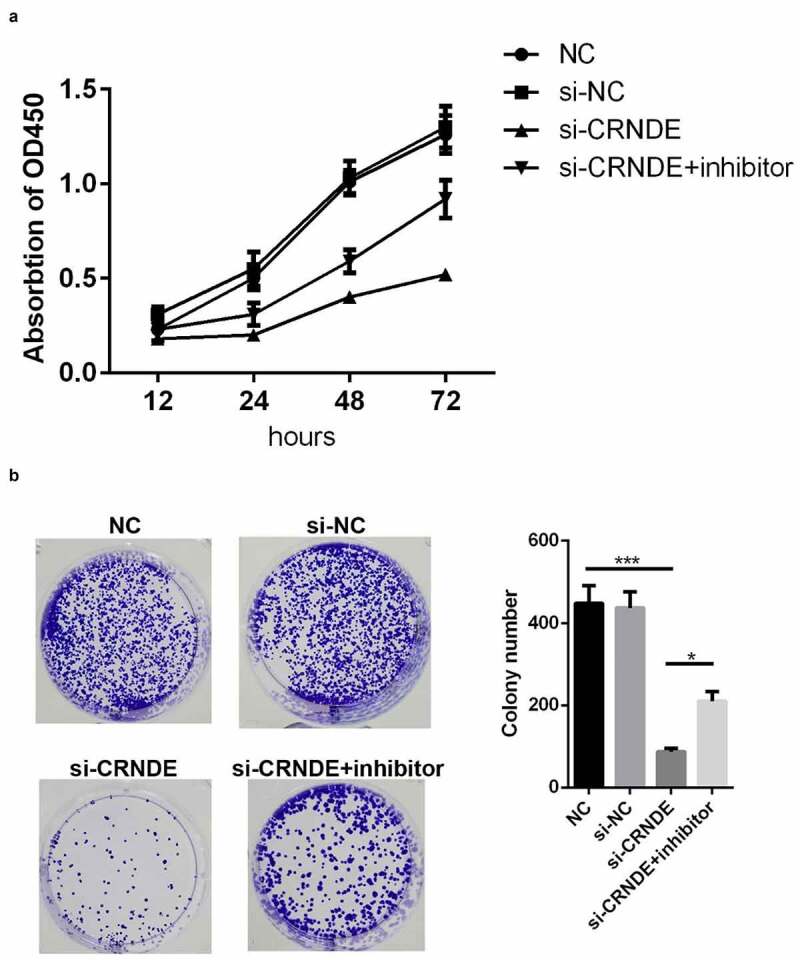
Knockdown of miR-146a-5p reversed the effects of si-CRNDE on the proliferation of PC. CCK-8(a) and colony formation (b) assays were used to detect the proliferation of PC3 cell lines. *p < 0.05, ***p < 0.001. Each experiment was repeated for 3 times

lncRNA CRNDE siRNA and miR-146a-5p inhibitor were used to establish stable lncRNA CRNDE–knockdown and miR-146a-5p–knockdown PC3 cells, and the efficiency of transfection was determined by qRT-PCR. The results showed that lncRNA CRNDE siRNA significantly inhibited the expression of lncRNA CRNDE (Figure 3a) and miR-146a-5p inhibitor decreased the levels of miR-146a-5p (Figure 3b). To evaluate the viability of PC3 cells transfected with siRNA lncRNA CRNDE, a CCK-8 assay was performed, with incubation periods of 0, 24, 48, or 72 h. As shown in Figure 4a, knockdown of CRNDE inhibited PC3 cell viability. Conversely, the transfection of the miR-146a-5p inhibitor partially mitigated the effects of lncRNA CRNDE (Figure 4a). Moreover, the results of colony formation analysis showed that lncRNA CRNDE siRNA inhibited the colony formation of PC3 cells and that miR-146a-5p inhibitor partially reversed the effects of lncRNA CRNDE siRNA (Figure 4b).

### Knockdown of lncRNA CRNDE induced apoptosis of PC cells

**Figure 5.**
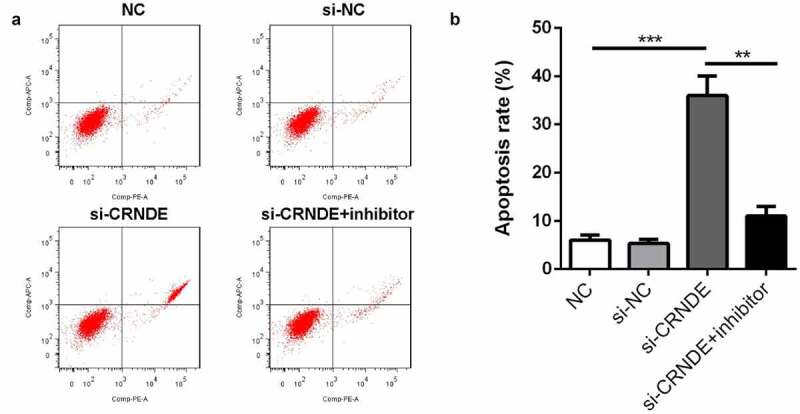
Knockdown of miR-146a-5p reversed the effect of si-CRNDE on the apoptosis rates of PC. (a-b) Flow cytometry assay was performed to determine the apoptosis rates of PC. **p < 0.01, ***p < 0.001. Each experiment was repeated for 3 times

To evaluate the effects of lncRNA CRNDE on PC3 cell apoptosis, a flow cytometry assay was employed. Figure 5 shows that the number of apoptotic cells in the siRNA CRNDE group was significantly increased compared to that in the siRNA NC group. The promotion of cell apoptosis by siRNA lncRNA CRNDE was partially reduced by the miR-146a-5p inhibitor.

### Role of lncRNA CRNDE in PC cell migration and invasion was mediated by miR-146a-5p

**Figure 6.**
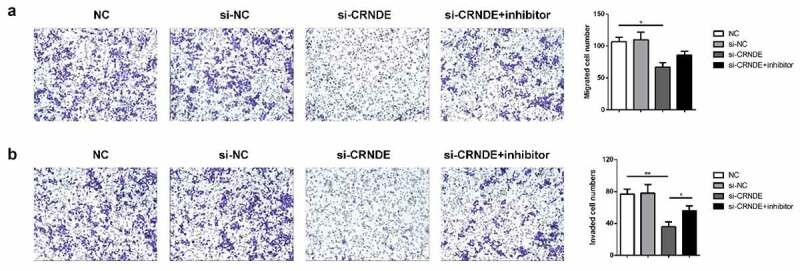
Knockdown of miR-146a-5p reversed the effect of si-CRNDE on the migration and invasion of PC. The number of migratory and invasive cells was determined by transwell chamber assays. Magnification ×200. **p < 0.01. Each experiment was repeated for 3 times

**Figure 7.**
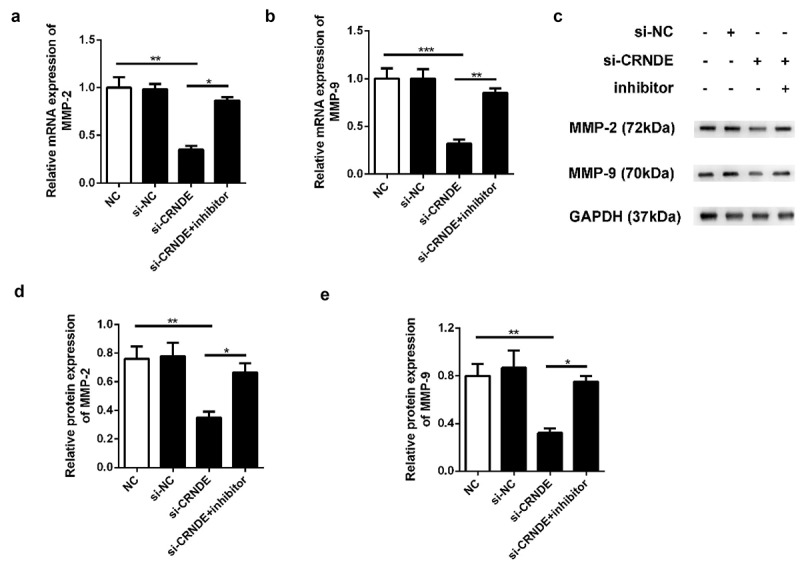
Knockdown of miR-146a-5p reversed the effect of si-CRNDE on the MMP-2 and MMP-9 expressions of PC. The qRT-PCR (a-b) and western blot (c-e) assays to detect the expressionS of MMP-2 and MMP-9 at mRNAs and proteins levels. **p < 0.01. Each experiment was repeated for 3 times

To determine whether miR-146a-5p mediated the functional role of lncRNA CRNDE in PC cells, we performed transwell chamber assays to evaluate the influence of lncRNA CRNDE on cell migration and invasion. We observed less migration and invasion among PC3 cells transfected with siRNA lncRNA CRNDE than among those transfected with siRNA NC (Figure 6a-b). Furthermore, the expression levels of metastasis-related mRNAs and proteins were evaluated using qRT-PCR and western blotting assays. As shown in Figure 7, downregulation of CRNDE expression resulted in decreased levels of MMP-2 and MMP-9 in the PC3 cell line. Knockdown of miR-146a-5p reversed the inhibition of MMP-2 and MMP-9 expression induced by siRNA lncRNA CRNDE at both the mRNA (Figure 7a-b) and protein (Figure 7c-e) levels. These findings indicated that knockdown of miR-146a-5p inhibited the migration and invasion of PC cells.

## Discussion

In recent years, several studies have proposed that dysregulation of lncRNAs contributes to PC carcinogenesis by affecting the biological behavior of cells. In the present study, we measured the expression of lncRNA CRNDE in PC cells to clarify its functional role in PC.

Numerous studies have demonstrated the involvement of lncRNAs in the tumorigenesis of bladder cancer [19], for which lncRNAs act as diagnostic and prognostic biomarkers [20]. For instance, Shan et al. reported that lncRNA NEAT1 contributes to bladder progression by regulating miR-410-mediated HMGB1, and Liu Y et al. demonstrated that lncRNA NNT-AS1 promotes the growth of bladder cancer cells by targeting the miR-1301-3p/PODXL axis and activating the Wnt pathway. In 2019, Fang C et al. revealed that DLX6-AS1 promotes cell growth and invasion in bladder cancer by modulating the miR-223-HSP90B1 axis [21–23]. Previous studies have shown that LINC00858, a novel lncRNA, is upregulated in several cancers and functions as a tumor promoter in colorectal cancer, non-small cell lung cancer, and osteosarcoma [24–26]. In these cancers, LINC00858 contributes to cell proliferation, migration, and invasion by acting as a competing endogenous RNA that binds to microRNAs. In a recent study, LncRNA CRNDE was found to acts as an oncogene in cervical cancer through sponging miR-183 to regulate CCNB1 expression [27]. However, the role of lncRNA CRNDE in PC remains unclear. In the present study, CRNDE was highly expressed in PC tissues. Furthermore, CRNDE promoted the proliferation, migration, and invasion of PC cells and inhibited PC cell apoptosis. Chen et al. also found that CRNDE regulate the growth and metastasis of PC via sponging miR-101 [28]. Tang et al. confirmed that CRNDE regulats the apoptosis and inflammation of pneumonia via targeting miR-141 [29]. These results were similar to ours.

Studies have increasingly revealed that miRNAs govern the biological functions of tumors during their development, including in PC. Wu et al. illustrated that knockdown of miR-139-5p302a increased the chemo‑resistance to paclitaxel in PC cells [30]. MiR-153 expression was also an independent prognostic factor in patients with PC, since miR-153-5p expression in PC tissues was associated with aggressive pathological parameters [31].Yang et al. illustrated that knockdown of miR-139-5p expression promotes the epithelial-mesenchymal transition process in PC progression [32]. Ji et al. demonstrated that miR-589-5p functions as a tumor inhibitor by regulating tumor migration, invasion, and apoptosis [33]. miR-877-5p has been widely studied in various cancers. In hepatocellular carcinoma (HCC), miR-877-5p targets cyclin-dependent kinase 14 to suppress the proliferation, migration, and invasion of HCC cells [34]. In glioblastoma, miR-877-5p regulated by lncRNA TRG-AS1 promotes tumor cell proliferation by targeting the suppressor of Zeste 12 [35]. Herein, we identified miR-146a-5p as a target of CRNDE in PC cells. Additionally, our rescue experiments showed that miR-146a-5p inhibitor partly mitigated the effect of siRNA lncRNA CRNDE on the malignant behavior of PC cells.

## Conclusion

In summary, our research offers original, significant insights into PC pathogenesis and a potential therapeutic strategy. We demonstrated that knockdown of lncRNA CRNDE suppressed miR-146a-5p expression, thereby inhibiting PC cell proliferation and metastasis and inducing apoptosis. This suggests that lncRNA CRNDE may be a novel therapeutic biomarker for PC.

## Data Availability

The datasets used and/or analyzed during the current study are available from the corresponding author on reasonable request.

## Highlights

- lncRNA CRNDE expression was upregulated in PC tissues
- lncRNA CRNDE knockdown suppressed PC cell proliferation and migration
- miR-146a-5p was identified as a direct target of lncRNA CRNDE
